# Production forecast for niger delta oil rim synthetic reservoirs

**DOI:** 10.1016/j.dib.2018.06.115

**Published:** 2018-07-04

**Authors:** Oluwasanmi A. Olabode, Gerald I. Egeonu, Ojo I. Temiloluwa, Oguntade Tomiwa, Bamigboye Oreofeoluwa

**Affiliations:** Department of Petroleum Engineering, Covenant University, P.M.B 1023 Ota Ogun State, Nigeria

**Keywords:** Reservoir Simulation, Design of Experiment, Placket Burman, Forecast, Exponential Decline, Monte-Carlo

## Abstract

The data sets in this article are related to a Placket Burman (PB) design of experiment (DOE) made on a wider range of uncertainties such as: reservoir, operational and reservoir architecture parameters that affect oil rim productivities. The design was based on a 2 level PB-DOE to create oil rim models which were developed into reservoir models using the Eclipse software and configured under the best depletion strategy of concurrent oil and gas production. Approximate solutions to the models was developed to forecast oil production using the least square method. The Monte-Carlo simulation approach was used in estimating 3 production forecasts for the oil rim reservoirs. This will help to create a probabilistic variety of forecasts that can further be used in making decisions.

**Specification Table**

**Table:** 

Subject area	*Petroleum Engineering*
More specific subject area	*Reservoir simulation/forecasting*
Type of data	*Tables and Figures.*
How data was acquired	*Oil rim reservoir parameters from the Niger-Delta*
Experimental features	*2 Level Placket Burman Design of Experiment*
Data source location	*Niger-Delta (Nigeria)*

**Value of data**

• This data incorporates a wider range of parameters such as reservoir architecture (dip), operational parameters (horizontal well length, horizontal well completion with respect to fluid contacts and well bore diameter) and extra reservoir parameters (oil density, bottom hole pressure and gas oil ratio constraints) in describing the nature of oil recovery in oil rim reservoirs.
• A response surface model can be developed from the given data to represent oil and gas recovery for all the models and a Pareto analysis can made to distinguish significant parameters that affect oil and gas recovery
• The models generated from the data can be used to derive decline curve equations using the linear regression method of an Excel Program from which probability production forecasts can be estimated using Monte-Carlo.
• The models generated from the data can also be classified based Pareto analysis ( large gas and large aquifer, small gas cap and small aquifer, large gas cap and small aquifer, large aquifer and small gas cap) and subjected to secondary and enhanced oil recovery schemes.
• A 3 level design of experiment can be carried out on the outcomes of the Pareto analysis to scientifically reduce quantify (and reduce where possible) uncertainties thus making the outcome more effective.

## 1 Data

Parameters affecting oil rims have been highlighted by Ref. [1] and validated by Ref. [2] and these are actually not adequate as some key parameters are often omitted. This inevitable affects the usefulness of the response surface models and effectiveness of the Pareto analysis [3], [4]. Table 1 show the range of uncertainties under a 2 level PB DOE used in the study. Table 2 describes the 2 level spatial distribution of uncertainties while Table 3 shows the PB DOE with the reservoir uncertainties. Models in Table 3 can be converted to reservoir models by incorporating Grid properties, PVT (Pressure, Volume and Temperature properties) and Saturation properties using the Schlumberger Reservoir Simulation software (Eclipse)

**Table 1:** Reservoir Uncertainties.

		**Parameter Range For The 15 uncertainties simulated**			
			**LOW**	**MID**	**HIGH**
	**Parameters**	**Units**	**−1**	**0**	**1**
1	Dip	degrees	1°	4	6
2	Gas Wetness	stb/Mscf	0.006	0.03	0.04
3	Oil Column Height	feet	20	40	70
4	M-factor(gas cap size)		0.7	3	6
5	Aquifer height to hydrocarbon thickness ratio(Aqfac)		0.7	3	6
6	Horizontal permeability (Kx, Ky)	mD	35	350	3500
7	Kv/Kh		0.001	0.01	0.1
8	Wellbore Diameter	feet	0.35	0.45	0.55
9	Oil Density	lb/cu. ft.	37	42	47
10	HGOC (Perforation with respect to the GOC)	feet	0.25	0.45	0.6
11	HWL (Horizontal well length)	feet	1200	1500	1800
12	Oil Rate	stb/day	1200	2200	3500
13	Krw (Rel. perm. to water)		0.2	0.35	0.6
14	GOR control (*Rsi)		2.5	5	7.5
15	BHP (Bottomhole Pressure)	psia	1500	1800	2200

**Table 2:** Placket–Burman design of experiment.

PLACKETT-BURMAN DESIGN OF EXPERIMENT (DOE) FOR 15 FACTORS															
The design is for 16 runs (the rows of dPB) manipulating 15 two-level factors (the last seven columns of dPB)															
The number of runs is a fraction 16/((2^15))=0. 00,048,828,125 of the runs required by a full factorial design.															
**Run No.**	**Dip**	**OGR**	**Ho**	**m-Factor**	**Aqfac**	**Kx, Ky**	**Kv/Kh**	**Bore Diam.**	**OIL DENSITY**	**HGOC**	**HWL**	**Qo**	**Krw**	GOR (*Rsi)	**BHP (psia)**
Model 1	1	1	1	1	1	1	1	1	1	1	1	1	1	1	1
Model 2	−1	1	−1	1	−1	1	−1	1	−1	1	−1	1	−1	1	−1
Model 3	1	−1	−1	1	1	−1	−1	1	1	−1	−1	1	1	−1	−1
Model 4	−1	−1	1	1	−1	−1	1	1	−1	−1	1	1	−1	−1	1
Model 5	1	1	1	−1	−1	−1	−1	1	1	1	1	−1	−1	−1	−1
Model 6	−1	1	−1	−1	1	−1	1	1	−1	1	−1	−1	1	−1	1
Model 7	1	−1	−1	−1	−1	1	1	1	1	−1	−1	−1	−1	1	1
Model 8	−1	−1	1	−1	1	1	−1	1	−1	−1	1	−1	1	1	−1
Model 9	1	1	1	1	1	1	1	−1	−1	−1	−1	−1	−1	−1	−1
Model 10	−1	1	−1	1	−1	1	−1	−1	1	−1	1	−1	1	−1	1
Model 11	1	−1	−1	1	1	−1	−1	−1	−1	1	1	−1	−1	1	1
Model 12	−1	−1	1	1	−1	−1	1	−1	1	1	−1	−1	1	1	−1
Model 13	1	1	1	−1	−1	−1	−1	−1	−1	−1	−1	1	1	1	1
Model 14	−1	1	−1	−1	1	−1	1	−1	1	−1	1	1	−1	1	−1
Model 15	1	−1	−1	−1	−1	1	1	−1	−1	1	1	1	1	−1	−1
Model 16	−1	−1	1	−1	1	1	−1	−1	1	1	−1	1	−1	−1	1
Model 17	−1	−1	−1	−1	−1	−1	−1	−1	−1	−1	−1	−1	−1	−1	−1
`Model 18	0	0	0	0	0	0	0	0	0	0	0	0	0	0	0

**Table 3:** Placket–Burman design of experiment with reservoir uncertainties.

**PLACKETT-BURMAN DESIGN OF EXPERIMENT (DOE) FOR 15 FACTORS**															
The design is for 16 runs (the rows of dPB) manipulating 15 two-level factors (the last seven columns of dPB)															
The number of runs is a fraction 16/((2^15))=0. 00,048,828,125 of the runs required by a full factorial design.															
Run No.	**Dip**	**OGR**	**Ho (ft.)**	**m-Factor**	**Aqfac**	**Kx, Ky**	**Kv/Kh**	**Bore Diam. (ft)**	**OIL DENSITY**	**HGOC (ft.)**	**HWL (ft.)**	**Qo**	**Krw**	GOR (*Rsi)	**BHP (psia0**
Model 1	6	0.04	70	6	6	3500	0.1	0.55	47	0.6	1800	3500	0.6	7.5	2200
Model 2	1	0.04	20	6	0.7	3500	0.001	0.55	37	0.6	1200	3500	0.2	7.5	1500
Model 3	6	0.006	20	6	6	35	0.001	0.55	47	0.25	1200	3500	0.6	2.5	1500
Model 4	1	0.006	70	6	0.7	35	0.1	0.55	37	0.25	1800	3500	0.2	2.5	2200
Model 5	6	0.04	70	0.7	0.7	35	0.001	0.55	47	0.6	1800	1200	0.2	2.5	1500
Model 6	1	0.04	20	0.7	6	35	0.1	0.55	37	0.6	1200	1200	0.6	2.5	2200
Model 7	6	0.006	20	0.7	0.7	3500	0.1	0.55	47	0.25	1200	1200	0.2	7.5	2200
Model 8	1	0.006	70	0.7	6	3500	0.001	0.55	37	0.25	1800	1200	0.6	7.5	1500
Model 9	6	0.04	70	6	6	3500	0.1	0.35	37	0.25	1200	1200	0.2	2.5	1500
Model 10	1	0.04	20	6	0.7	3500	0.001	0.35	47	0.25	1800	1200	0.6	2.5	2200
Model 11	6	0.006	20	6	6	35	0.001	0.35	37	0.6	1800	1200	0.2	7.5	2200
Model 12	1	0.006	70	6	0.7	35	0.1	0.35	47	0.6	1200	1200	0.6	7.5	1500
Model 13	6	0.04	70	0.7	0.7	35	0.001	0.35	37	0.25	1200	3500	0.6	7.5	2200
Model 14	1	0.04	20	0.7	6	35	0.1	0.35	47	0.25	1800	3500	0.2	7.5	1500
Model 15	6	0.006	20	0.7	0.7	3500	0.1	0.35	37	0.6	1800	3500	0.6	2.5	1500
Model 16	1	0.006	70	0.7	6	3500	0.001	0.35	47	0.6	1200	3500	0.2	2.5	2200
Model 17	1	0.006	20	0.7	0.7	35	0.001	0.35	37	0.25	1200	1200	0.2	2.5	1500
Model 18	4	0.03	40	3	3	350	0.01	0.45	42	0.45	1500	2200	0.35	5	1800

## 2 Experimental design, materials and methods

### 2.1 Formulation of approximate model

**Fig. 1:**
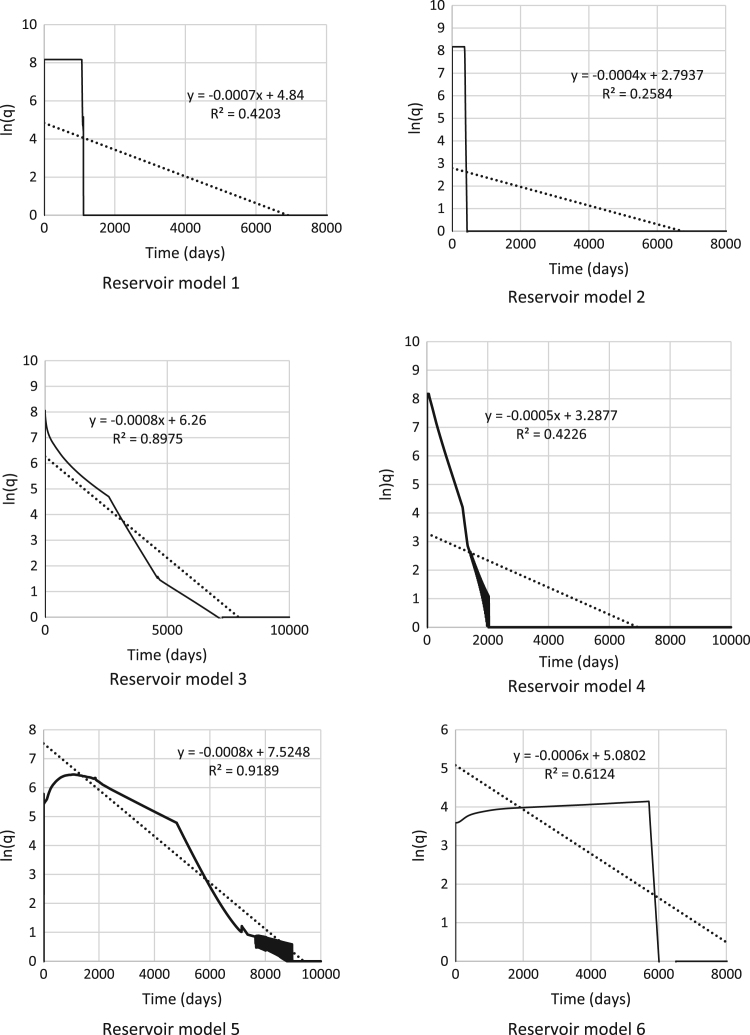
*R*^2^ values and production profile equations.

Fig. 1 shows the *R*^2^ values and profile equations to the production profiles for some of the models were used to develop decline curve based models. Table 4 shows the original fluids in place, fluids produced and recovery factors under a concurrent oil and gas production. These models are a special form of response surface models using the least square method. The initial stages of production were considered in the proxy equation and further generalized to obtain 3 production forecast models using the exponential decline curve model defined by Ref. [5] in Eq. (1).

(1) $$N_{p}\left(t\right)=\frac{q^{*}}{D}\left(e^{{-\mathrm{Dt}}_{p}}-e^{-\mathrm{Dt}}\right)+q_{i}t_{p}$$

**Table 4:** Oil and gas production profile.

**Cumulative oil production**						**Cumulative gas production**				
**Model no**	**CUMM. PROD. (stb)**	**OIIP (Mstb)**	**RF (%)**	**OCIP (Mstb)**	**NFA**	**GIIP (Mscf)**	**CUMM. PROD. (Mscf)**	**RF (%)**	**GCIP (Mscf)**	**NFA**
**model 1**	3780,909	26,905	14	23,124	1095	589,284	277,540	47.1	311,744	1055
**Model 2**	1313,602	8471	16	7157	376	286,844	198,933	62.9	87,911	376
**Model 3**	1222,449	55,616	22	4339	3995	315,410	203,742	64.6	111,668	3960
**Model 4**	299,295	28,071	1	27,773	1609	330,684	50,000	15.1	280,684	1410
**Model 5**	1,859,613	16,267	11	14,408	6204	96,761	63,316	65.4	33,445	6000
**Model 6**	316,218	4690	7	4374	6000	62,184	28,892	46.5	33,292	6000
**Model 7**	914,593	5750	16	4836	740	412,231	152,432	37.0	259,799	740
**Model 8**	1,208,603	23,521	5	22,312	1000	42,015	22,561	53.7	19,454	1128
**Model 9**	10,750,810	60,981	18	50,230	8672	1,009,138	620,356	61.5	388,782	8660
**Model 10**	1,284,098	4542	28	3258	1069	122,448	49,020	40.0	73,428	1069
**Model 11**	387,335	4161	9	3773	7990	135,949	64,556	47.5	71,393	8000
**Model 12**	1,971,314	25,498	8	23,527	4000	612,799	445,696	62.5	167,103	4000
**Model 13**	1,154,199	17,063	7	15,909	5793	97,566	37,710	38.7	59,856	6000
**Model 14**	248,143	2878	9	2630	270	20,356	12,944	63.6	7412	542
**Model 15**	191,164	2728	7	2549	70.5	65,116	31,860	48.9	33,256	70.5
**Model 16**	1,499,402	33,279	7	21,631	470	68,235	27,672	40.6	40,563	470
**Model 17**	88,231	3495	3	3406	3000	20,049	12,999	64.8	7050	3000
**Model 18**	457,304	11,989	4	11,532	1000	132,143	50,000	37.8	82,143	10,000

*Where OOIP is oil initially in place, OCIP is oil currently in place, GIIP is gas initially in place, GCIP is gas currently in place and NFA means no further action.

With the linear regression method of an Excel program, the calculations of the continuous decline rate constant, *R*-squared value of the straight line fitting, the production rate,∗, when the straight line is extrapolated to time zero were estimated.

**Fig. 2:**
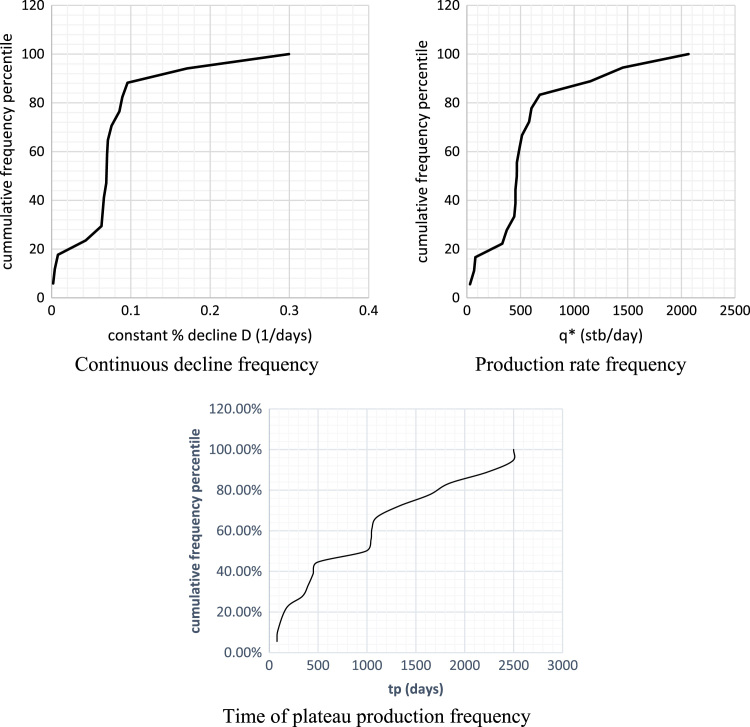
Cumulative frequency plots.

The time of plateau production, initial production rates *q*^⁎^ and continuous decline rate constants used in analyzing the decline plots are shown in Table 5, Table 6, Table 7 while Fig. 2 presents the plot.

**Table 5:** Time of plateau production cumulative frequency.

tp (days)	% Cumm. *F*	*F*	Cumm. *F*
80	5.56	1	1
90	11.11	1	2
180	22.22	2	4
340	27.78	1	5
400	33.33	1	6
450	38.89	1	7
490	44.44	1	8
990	50.00	1	9
1041	55.56	1	10
1051	61.11	1	11
1100	66.67	1	12
1330	72.22	1	13
1640	77.78	1	14
1840	83.33	1	15
2230	88.89	1	16
2490	94.44	1	17
2500	100.00	1	18

**Table 6:** Production rate cumulative frequency.

Q* (stb/day)	% Cumm. *F*	*F*	Cumm. *F*
28.623	5.5555556	1	1
67.235	11.111111	1	2
78.901	16.666667	1	3
329.9	22.222222	1	4
371.22	27.777778	1	5
441.89	33.333333	1	6
452.55	38.888889	1	7
452.55	44.444444	1	8
465.72	50	1	9
466.49	55.555556	1	10
489.01	61.111111	1	11
513.26	66.666667	1	12
579.86	72.222222	1	13
602.09	77.777778	1	14
679.33	83.333333	1	15
1152	88.888889	1	16
1453.8	94.444444	1	17
2066.7	100	1	18

**Table 7:** Cumulative frequency constant decline D (1/days).

Constant % decline D (1/days)	% Cumm. *F*	F	Cumm. *F*
0.002	5.88235294	1	1
0.004	11.7647059	1	2
0.008	17.6470588	1	3
0.043	23.5294118	1	4
0.063	29.4117647	1	5
0.066	41.1764706	2	7
0.069	47.0588235	1	8
0.07	58.8235294	2	10
0.071	64.7058824	1	11
0.076	70.5882353	1	12
0.086	76.4705882	1	13
0.089	82.3529412	1	14
0.096	88.2352941	1	15
0.171	94.1176471	1	16
0.299	100	1	17

**Fig. 3:**
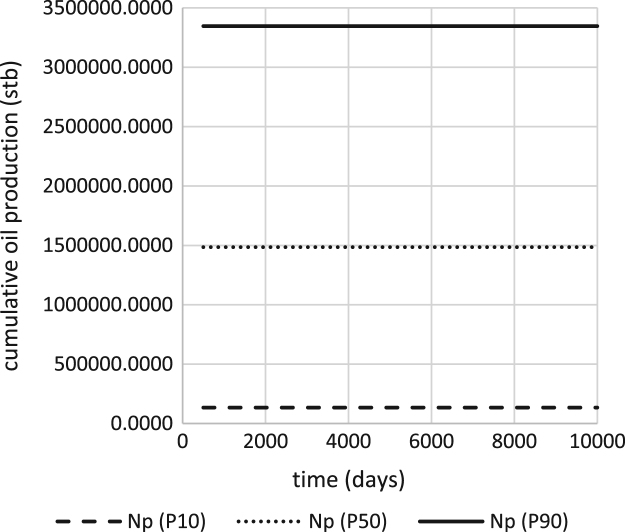
Probabilistic Production Forecast for oil rate of 1800 stb/d.

The values of the selected models are shown in Table 8 and were used to generate the probabilistic range of production forecast (Fig. 3) at 1500 stb/day.

**Table 8:** Probability Distribution of the Input Variables of the Proxy Equation.

Percentile	q* (stb/day)	*D* (1/Days)	tp (days)
P10	1.94075	0.003	89
P50	465.72	0.073	990
P90	1352.248	0.0961	2231
