# Supplementary material for: Production forecast for niger delta oil rim synthetic reservoirs
Source: Data Brief. 2018 Jul 4;19:2205–14. doi: 10.1016/j.dib.2018.06.115 (PMC6141438; doi:10.1016/j.dib.2018.06.115)
Supplement: Supplementary file 1 — Supplementary material [file mmc1.docx]

**Conflict of interest**

All the author confirms as No conflict of interest.
